# Comparison of CO_2_ Laser and Microdebrider in the Surgical Treatment of Pediatric Recurrent Respiratory Papillomatosis: A Retrospective Analysis

**DOI:** 10.3390/bioengineering13060713

**Published:** 2026-06-22

**Authors:** Kadylova Yerkezhan, Nazym S. Sagandykova, Madina Baurzhan, Aigerim Mashekova, Bekpan Almat, Autalipov Darkhan, Olzhas Mukhmetov, Damir Abdrakhmanov, Eddie Yin Kwee Ng, Sayagul Kairgeldina

**Affiliations:** 1 Corporate Fund “University Medical Center”, Astana 010000, Kazakhstan; erkejan9898@mail.ru (K.Y.); doctor.ent.alm@gmail.com (N.S.S.); almat-bekpanov@mail.ru (B.A.); darkhanautalipov88@gmail.com (A.D.); 2 Scientific Research Institute of Balneology and Medical Rehabilitation, Astana 010000, Kazakhstan; madina_baurzhan@mail.ru (M.B.); sanborovoe@mail.ru (D.A.); sanperevod@mail.ru (S.K.); 3 School of Intelligent Systems, Astana IT University, Astana 010000, Kazakhstan; olzhas.mukhmetov@nu.edu.kz; 4 School of Mechanical and Aerospace Engineering, Nanyang Technological University, Singapore 639798, Singapore

**Keywords:** laryngeal papillomatosis, recurrent respiratory papillomatosis, children, CO_2_ laser, microdebrider

## Abstract

**Background**. Recurrent respiratory papillomatosis (RRP) in children remains a pressing issue in pediatric otolaryngology, characterized by a chronic course, frequent relapses, and the need for repeat surgical interventions. The aim of this study was to evaluate whether the surgical technique used for primary removal of pediatric RRP—CO_2_ laser, microdebrider, or a combined approach—was associated with clinically documented recurrence and early recurrence within 12 months. **Materials and Methods**. A retrospective study of 53 medical records of children who underwent their first surgery for RRP between 2019 and 2023 was conducted. Three surgical approaches were used: CO_2_ laser, microdebrider, and a combined method. Statistical analysis was performed using Pearson’s χ^2^ test, and the strength of association was evaluated with Cramér’s V. **Results**. The most frequently used method was the CO_2_ laser (*n* = 25), followed by a microdebrider (*n* = 16), and a combined method (*n* = 12). During the observation period, disease recurrence was recorded in 35 of 53 patients (66.0%): in 20 children, within the first 12 months after surgery, and in 15, after more than 12 months. No recurrence was documented in the available medical records for 18 patients during the observation period. No statistically significant effect of the surgical treatment method on the recurrence rate (*p* = 0.813) or the risk of early recurrence (*p* = 0.926) was found. Also, no significant association was found between the child’s age and either the overall recurrence rate (*p* = 0.510) or the likelihood of early recurrence (*p* = 0.217). **Conclusions**. Within the limitations of this retrospective single-center study, neither the surgical treatment method nor the patient’s age was associated with clinically documented recurrence or early recurrence recorded in the available medical records.

## 1. Introduction

Recurrent respiratory papillomatosis (RRP) is a chronic disease associated with human papillomavirus (HPV), primarily types 6 and 11. It is characterized by the formation of papillomatous growths in the respiratory tract, mostly in the larynx [1,2,3]. This pathology is of particular importance in pediatric practice, as even limited papilloma growth in children can lead to airway obstruction due to the anatomical narrowness of the larynx. Clinically, the disease can present with hoarseness, dysphonia, stridor, and difficulty breathing, and in severe cases, may require urgent airway management or even tracheostomy [1,4].

The juvenile form of RRP is typically diagnosed in early childhood and often has a recurrent course. An earlier age at disease onset is associated with a more aggressive clinical course, more frequent surgical interventions, shorter intervals between surgeries, and potential dissemination to the lower respiratory tract [3,5,6,7,8,9]. Therefore, recurrence after primary surgical treatment is one of the most significant clinical outcomes for both the physician and the patient and their family.

Currently, there is no standardized drug therapy for the curative treatment of RRP in children. Various adjuvant approaches have been described in the literature, including bevacizumab, cidofovir, interferon-α, HPV vaccination, and new immunotherapeutic strategies [10,11,12,13,14]. However, these methods are generally considered adjunctive or disease-modifying rather than a complete alternative to surgical treatment. Therefore, surgical removal of papillomas remains the primary treatment for children with RRP [5,12,13,14].

Among the most common surgical methods for treating RRP are CO_2_ laser ablation and microdebrider-assisted papilloma removal [12,13,14,15,16,17,18]. These approaches differ not only in their technical execution but also in their mechanism of action on tissue. The CO_2_ laser provides highly precise thermal ablation of pathological tissue and good intraoperative hemostasis; however, its use may be associated with the risk of thermal damage to the surrounding mucosa, scarring, adhesion formation, or laryngeal membrane rupture. A microdebrider, in contrast, mechanically removes papillomatous tissue with simultaneous aspiration, which can improve visualization of the surgical field, shorten the duration of the procedure, and reduce thermal impact on surrounding tissue [16,17].

Despite the widespread use of both methods, the question of whether the choice of surgical technique during initial intervention influences the subsequent course of the disease remains poorly understood. In real-world clinical practice, the choice between a CO_2_ laser, a microdebrider, or a combined approach is often determined by the facility’s equipment, the surgeon’s experience, the extent of the lesion, and local clinical preferences. However, from a long-term patient management perspective, not only the technical convenience of the method is key, but also its potential relationship with the rate of subsequent recurrences.

Most published studies focus on technical and perioperative parameters: surgical duration, severity of bleeding, quality of visualization of the surgical field, postoperative edema, and safety of the procedure [12,13,14,16,17,19]. These parameters are certainly important, but they do not fully reflect the chronic and recurrent nature of RRP. For children and their families, the most significant issues remain the recurrence of upper airway papillomas, the timing of recurrence, and the need for repeat surgical treatment.

Given this, a direct comparison of the most commonly used surgical techniques is of practical interest. The aim of this study was to evaluate the association between the surgical technique used for primary removal of RRP of upper airways in children—CO_2_ laser, microdebrider, or a combined approach—and clinically documented recurrence, including early recurrence within 12 months, based on available medical records.

## 2. Related Work

CO_2_ laser ablation and microdebrider-assisted papilloma removal have been described in the literature as some of the main surgical approaches for the treatment of recurrent respiratory papillomatosis, including the juvenile form of the disease [13,14,16,17,18]. Both methods aim to reduce the volume of papillomatous tissue and restore airway patency, but differ in their mode of action, technical capabilities, and potential impact on laryngeal tissue.

The CO_2_ laser is traditionally used in laryngeal surgery due to its high precision and the ability to control bleeding in a limited surgical field. However, the use of thermal energy is associated with the risk of damage to the surrounding mucosa, scarring, adhesions, and laryngeal membranes [12,17]. These complications are particularly significant in pediatric practice, as even mild airway narrowing in a child can have significant clinical consequences.

A microdebrider is another widely used method for surgical removal of papillomas. It provides mechanical tissue resection with simultaneous aspiration, which can improve visibility of the surgical field and reduce the duration of the procedure. Several studies have noted that a microdebrider can be particularly useful for removing large papillomatous growths and may be associated with less postoperative swelling compared to thermal-based methods [16,17]. However, it remains unclear whether these technical advantages translate into better recurrence control.

Comparative data on the efficacy of the CO_2_ laser and the microdebrider remain limited and inconsistent. A study by Rashid et al. demonstrated certain advantages of a microdebrider in the treatment of juvenile RRP [16]. At the same time, systematic reviews and other publications highlight significant variability in the available data: studies differ in patient age, disease severity, prior treatment history, follow-up duration, use of adjuvant therapy, and endpoint definitions [12,13,14,17]. This complicates drawing a definitive conclusion about the superiority of any single method for recurrence prevention.

A separate problem is that many studies primarily evaluate perioperative parameters, while recurrence after primary intervention is less frequently considered. Surgical duration, bleeding, imaging, and the severity of postoperative edema are important for assessing the safety and technical efficacy of a method, but they do not fully address the key clinical question: whether a child will experience papilloma regrowth, and if so, when will it occur?

Other surgical techniques, including coblation, are also discussed in the literature. Recent pediatric data suggest comparable efficacy and safety between coblation and microdebriders; however, convincing evidence of a clear advantage of coblation in reducing recurrence rates has not yet been obtained [19]. Adjuvant and non-surgical strategies, including bevacizumab, HPV vaccination, and immunotherapeutic approaches, are also being actively studied [10,11,20,21]. However, in most cases, they are considered adjunctive disease control methods rather than a replacement for surgical removal of papillomas, especially in pediatric practice.

Thus, a practical clinical question remains: is the surgical technique chosen for the initial treatment of RRP in children associated with a subsequent risk of clinically documented recurrence? This study does not aim to introduce a new surgical technology. Its significance lies in the comparison of three real-world approaches—CO_2_ laser, microdebrider, and a combined method—in a single-center pediatric cohort. Separate assessment of overall clinically documented relapse and early relapse within 12 months allows us to supplement the existing data and bring them closer to the conditions of everyday clinical practice.

## 3. Materials and Methods

*Study design*. Retrospective case series with comparative groups.

*Study Population*. The study analyzed the medical records of patients treated in Pediatric Surgery Department No. 2 of the University Medical Center of the Corporate Fund of Astana, Republic of Kazakhstan, from January 2019 to December 2023.

The study included pediatric patients with RRP who underwent first-time inpatient surgical treatment for laryngeal papillomatosis. Papillomatous lesions were predominantly localized on the vocal folds, with possible extension to the supraglottic and subglottic regions. All patients presented with clinical signs such as respiratory dysfunction and voice changes.

Inclusion criteria: children under 18 years of age; newly diagnosed laryngeal and tracheal papillomatosis; exclusion criteria: readmission; history of surgical treatment or any medication; presence of an acute upper airway infection; other reasons of stenosis (tumors, cicatricial stenosis).

The diagnosis was made based on clinical presentation and endoscopic fibrolaryngoscopy, and confirmed by histopathological examination.

**Figure 1:**
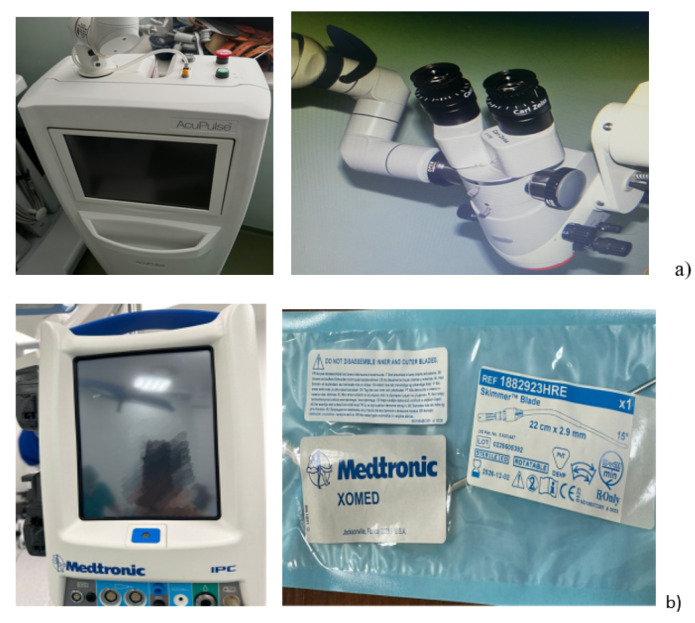
Appearance of the CO_2_ surgical laser system AcuPulse (**a**) and the Straightshot^®^ M4 microdebrider (**b**), used for endolaryngeal removal of papillomatosis.

Surgical treatment methods. Papillomatosis was treated using three main surgical approaches: CO_2_ laser vaporization or ablation, mechanical resection with a microdebrider, and a combination of both techniques. The choice of surgical method was determined by the availability of medical equipment at the time of the patient’s hospitalization. Direct surgical laryngoscopes for microscopic laryngeal surgery, an operating microscope and endoscope, and a microdebrider system were used during the surgeries (Figure 1).

Information on the technical equipment of the operating units in clinics performing pediatric laryngeal surgery is presented in Table 1.

Recurrence was assessed retrospectively using medical records from our center. Repeat presentation was usually initiated by patients or their caregivers because of symptoms such as breathing difficulty, voice changes, or hoarseness. These symptoms served as indications for repeat laryngeal endoscopy and hospitalization. Early recurrence was defined as recurrence documented within 12 months after surgery. Telephone follow-up was not performed. Patients who did not return to our center were classified as having no documented recurrence in our records, but not necessarily as recurrence-free.

*Statistical analysis*. Statistical data processing was performed using categorical variable analysis methods. All qualitative indicators are presented as absolute values and proportions—*n* (%). The Pearson chi-square (χ^2^) test was used to assess the association between surgical treatment method and recurrence. This same test was used to analyze the impact of surgical method on the development of early recurrence, as well as to assess the relationship between the child’s age, recurrence rate, and the risk of early recurrence. The Cramér’s V coefficient was calculated to determine the strength of the association. Differences were considered statistically significant at *p* < 0.05.

## 4. Results

**Figure 2:**
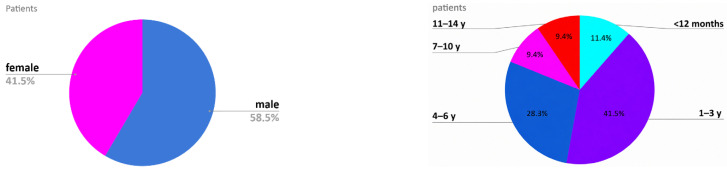
Distribution of pediatric patients with RRP by sex and age (%).

*Population characteristics*. Between 2019 and 2023, the medical records of 53 patients with laryngeal papillomatosis who underwent inpatient surgery for the first time were analyzed. The age and sex distribution of the patients is shown in Figure 2.

Age groups were determined in accordance with WHO recommendations: up to 12 months—6 patients (11.4%); 1–3 years (early childhood)—22 patients (41.5%); 4–6 years (preschool age)—15 patients (28.3%); 7–10 years (primary school age)—5 patients (9.4%); 11–14 years (adolescence)—5 patients (9.4%); and 15–18 years—no patients were registered [17,18]. Due to the limited sample size and the small number of observations in several age categories, these groups were subsequently consolidated into three broader categories for the statistical analysis.

*Results of surgical treatment*. The most frequently used method was the CO_2_ laser (*n* = 25), followed by a microdebrider (*n* = 16) and a combined method (CO_2_ + microdebrider, *n* = 12). Detailed information on the annual distribution of children from different age groups by three types of surgery during 2019–2023 is provided in Appendix A and Appendix B.

Data analysis showed that recurrence occurred after all three methods of laryngeal papillomatosis removal (Table 2). Over the observation period, 35 of 53 patients (66.0%) who underwent surgical treatment were readmitted to the department because of disease recurrence. The number of patients who developed recurrence within the first year after surgery was slightly higher than the number of those who relapsed more than 12 months after surgery (20 vs. 15). Among children without recurrence, the largest proportion underwent CO_2_ laser surgery (44.4%). Likewise, among patients with recurrence, this method was also the most common (48.5%).

**Figure 3:**
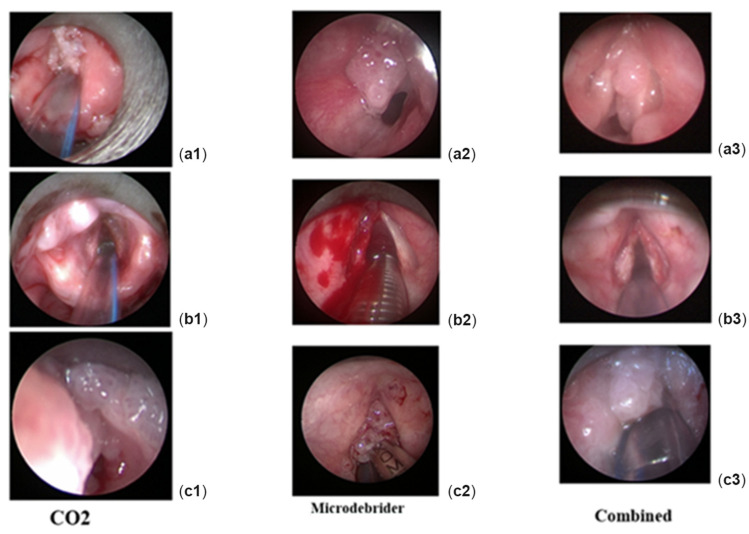
Representative endoscopic images obtained before surgery (**a1**–**a3**), immediately after papilloma removal (**b1**–**b3**), and at documented recurrence (**c1**–**c3**). Columns represent the CO_2_ laser, microdebrider, and combined-treatment groups, respectively.

Figure 3 presents endoscopic images of the larynx before surgery, after removal of papillomatous lesions, and during subsequent disease recurrence in the CO_2_ laser, microdebrider, and combined treatment groups. In all three groups, postoperative images confirmed successful removal of the pathological lesions and restoration of the glottic lumen. Nevertheless, recurrence was observed following each of the studied methods, indicating a persistent risk of papilloma regrowth irrespective of the surgical technique applied.

*Statistical analyses*. Statistical analysis revealed no significant association between the surgical treatment method and the recurrence rate of LP (χ^2^ = 0.413; *p* = 0.813), or between the surgical method and the risk of early recurrence within the first 12 months after surgery (χ^2^ = 0.154; *p* = 0.926). The effect size in both cases was minimal (Cramér’s V = 0.088 and 0.054, respectively), indicating the absence of a clinically significant effect of the surgical method on these outcomes.

No statistically significant association was found between the child’s age (Table 3) and the development of recurrence (χ^2^ = 1.35; df = 2; *p* = 0.510). No significant effect of age on the risk of early recurrence within the first 12 months after surgery was also found (χ^2^ = 3.05; df = 2; *p* = 0.217). The effect size in both cases was weak (Cramér’s V = 0.16 and 0.24, respectively), indicating the absence of a significant effect of age on these outcomes in the study sample.

## 5. Discussion

This study analyzed the medical records of 53 children with RRP treated in the department and compared the effects of CO_2_ laser and microdebrider surgery on clinically documented recurrence and early recurrence within 12 months.

Our analysis yielded several key findings. No statistically significant association was identified between the choice of surgical method and the recurrence rate (*p* = 0.813), or between the surgical method and the rate of early recurrence within 12 months after treatment (*p* = 0.926). In addition, patient age was not associated with recurrence regardless of the type of intervention used (*p* = 0.510). Most of the children included in the study were aged 1–6 years, and this age group accounted for 82.9% of all children who experienced recurrence. Although most recurrences were observed in younger children, no statistically significant association between age and recurrence was identified in our sample.

Our findings are consistent with previously published studies in which CO_2_ laser and microdebrider techniques are regarded as the main modern surgical approaches for the treatment of RRP. In particular, the study by [16] reported no statistically significant differences in recurrence rates between these methods. Likewise, Torres-Canchala et al. [11] did not demonstrate any significant long-term advantage of either approach in reducing the risk of disease recurrence. Derkay et al. [22] showed that the use of a microdebrider reduces operative time and intraoperative blood loss, but does not significantly affect recurrence rates. Similarly, Afridi et al. [23] noted that adjuvant therapy, when used alone, is not superior to surgical treatment in terms of effectiveness and may, in some cases, even negatively affect outcomes. Despite the range of proposed adjuvant therapeutic approaches, their clinical efficacy remains variable, and no unified treatment strategy has yet been established [5].

It should also be noted that, unlike our study, a number of published works have evaluated other surgical technologies, including coblation. One of the advantages of this method is its ability to perform ablation, coagulation, aspiration, and irrigation simultaneously using a single instrument, which may reduce blood loss and improve visualization of the surgical field. However, Donne et al. [24] and McNally et al. [25] found no statistically significant differences between coblation and microdebrider use in the interval between repeat surgeries, suggesting comparable recurrence rates.

In recent years, interest in immunotherapeutic approaches targeting key pathogenetic mechanisms of RRP has increased considerably. New immunotherapies are currently under development that may enhance the cellular immune response against HPV types 6 and 11. For example, clinical trials of INO-3107 demonstrated enhancement of the specific CD4 and CD8 T-cell response against HPV, which may help reduce the need for repeat surgery [25]. Similar findings were reported in a study of the gene therapy PRGN-2012 [19]. One of the most promising directions is the development of the innovative gene therapy drug Papzimeos, which targets HPV 6 and 11. This drug is considered the first potentially etiotropic approach capable of reducing patients’ dependence on repeated surgical interventions and providing long-term remission in some cases [23].

Age also appears to play an important role in the course of the disease. According to Oh et al. [6] and Lepine P et al. [20], early disease onset, particularly before the age of 5 years, is associated with a more aggressive clinical course. A similar view is reflected in the Chinese clinical guidelines, which note that early manifestation of the disease is often accompanied by frequent recurrences and a high number of repeat surgeries [21].

At present, HPV vaccination is considered an important component in the prevention of recurrent respiratory papillomatosis. Research findings indicate that in countries with high vaccination coverage, particularly Australia, the incidence of RRP has significantly decreased [13]. Systematic reviews also confirm that vaccination contributes to reducing mother-to-child transmission of the virus and lowers the risk of disease development [25]. However, vaccination does not provide a complete cure and is currently regarded primarily as a preventive strategy [14]. Some studies also suggest that it may help attenuate the course of the disease [25].

Currently, there is no approved standardized medical treatment for RRP. This confirms that surgical treatment remains predominantly symptomatic in most cases and does not completely prevent recurrence.

Several limitations must be considered when interpreting the results of this study. First, the study had a retrospective design and was based on a review of available medical records. The relatively small sample size may also have reduced the statistical power of the study and limited the ability to detect significant differences between groups.

An important limitation is the method used to assess recurrence. Recurrence was determined solely based on patient return visits to our center, repeat endoscopy, and subsequent histological verification. Telephone follow-up was not conducted, and data from other medical institutions were unavailable. Therefore, patients who did not return to our center during the observation period were classified as having no documented recurrence in our medical records. However, this does not completely exclude the possibility of recurrence diagnosed or treated at another institution. Therefore, our results reflect the frequency of clinically documented recurrences at our center, rather than the absolute recurrence rate in the study population.

Furthermore, postoperative complications and adverse events were not systematically assessed when collecting data from patient records. Therefore, a reliable comparison of the safety profile between the CO_2_ laser, microdebrider, and the combined method was not possible in this study. Uneven distribution of patients by age group and surgical treatment method may also have influenced the results of the subgroup analysis. The choice of surgical technique depended on equipment availability, clinical situation, and institutional practice, which could have introduced selection bias.

Also, this study did not thoroughly analyze several potentially significant clinical factors, including disease severity, anatomical extent of papillomatous lesions, HPV type, postoperative follow-up, and the use of adjuvant therapy. Given that a change in surgical instrumentation alone may not have a significant impact on the risk of recurrence in RRP, future studies should consider not only the choice of surgical technique but also combined treatment strategies, including adjuvant approaches such as bevacizumab, HPV vaccination, and other modern methods aimed at reducing the rate of reoperations.

Thus, the data obtained should be considered the results of a single-center retrospective study reflecting real-world clinical practice but have limited external validity. Larger prospective studies with long-term follow-up, standardized assessment of recurrence, complications, and clinical severity are needed to more accurately assess the impact of surgical technique and adjuvant therapy on the course of RRP in children.

## 6. Conclusions

In the present study, no statistically significant association was found between surgical treatment method and RRP recurrence in children (χ^2^ = 0.413; df = 2; *p* = 0.813; Cramér’s V = 0.088) or the risk of early recurrence within the first 12 months after surgery (χ^2^ = 0.154; df = 2; *p* = 0.926; Cramér’s V = 0.054). In addition, the child’s age did not demonstrate a significant association with either the overall recurrence rate (χ^2^ = 1.35; df = 2; *p* = 0.510; Cramér’s V = 0.16) or the likelihood of early recurrence (χ^2^ = 3.05; df = 2; *p* = 0.217; Cramér’s V = 0.24). Thus, within the study sample, neither surgical treatment method nor patient age were associated with the outcomes studied. However, the results obtained should be interpreted with caution due to the limited sample size and small subgroup sizes, requiring further research on larger samples.

## Figures and Tables

**Table 1:** Technical equipment of the CF “University Medical Center” for surgical treatment of RRP.

Equipment/Tool	Model/Brand	Country/Manufacturer	Application	Notes
Direct surgical laryngoscope	Karl Storz	Tuttlingen, Germany	Visual access to the larynx	For laryngoscopic surgery
Surgical microscope	Zeiss surgical microscope	Carl Zeiss, Oberkochen, Germany	Carrying out the operation with high precision	Enlargement of laryngeal tissue
Microdebrider	Straightshot^®^ M4 Microdebrider	Medtronic, Minneapolis, MN, USA	Mechanical removal of papillomas	Used in conjunction with the Skimmer Tricut blades
CO_2_ laser	CO_2_ surgical laser system AcuPulse Series S16	Lumenis Ltd., Yokneam, Israel	Ablation (vaporization) of papillomas	High power laser, minimal bleeding, minimal damage to surrounding tissues

**Table 2:** Distribution of patients by surgical treatment methods and recurrence rate.

Surgical Method	Total Patients *n* (%)	Early Recurrence (<12 Months), *n* (%)	Late Recurrence (>12 Months), *n* (%)	No Documented Recurrence, *n* (%)
Microdebrider	16 (30.2%)	6 (30%)	5 (33.3%)	5 (27.8%)
CO_2_ laser	25 (47.2%)	10 (50.0%)	7 (46.7%)	8 (44.4%)
CO_2_ + Microdebrider	12 (22.6%)	4 (20%)	3 (20.0%)	5 (27.8%)

**Table 3:** Distribution of patients by age group, surgical method, and recurrence outcome.

Age Group	CO_2_ Laser—Early Recurrence	CO_2_ Laser—Late Recurrence	CO_2_ Laser No Recurrence	Microdebrider—Early Recurrence	Microdebrider—Late Recurrence	Microdebrider No Recurrence	Combined—Early Recurrence	Combined—Late Recurrence	Combined—No Recurrence
<3 y	3	4	5	4	3	4	2	1	2
4–6 y	4	0	1	2	2	0	1	1	2
7–18 y	2	2	2	0	0	1	1	1	1
Total	10	7	8	6	5	5	4	3	5

## Data Availability

Dataset available on request from the authors.

## Appendix A

**Table A1:** Distribution of patients by age group and surgical treatment methods (2019–2023).

Year	Surgical Method	<12 Months *n* (%)	12 Months–3 y *n* (%)	4–6 y *n* (%)	7–10 y *n* (%)	11–14 y *n* (%)	15–18 y *n* (%)
2019	CO_2_ laser	0 (0%)	1 (1.9%)	0 (0%)	2 (3.8%)	3 (5.7%)	0 (0%)
	Microdebrider	0 (0%)	0 (0%)	1 (1.9%)	0 (0%)	0 (0%)	0 (0%)
	Combined (CO_2_ + Microdebrider)	0 (0%)	0 (0%)	1 (1.9%)	1 (1.9%)	0 (0%)	0 (0%)
2020	CO_2_ laser	2 (3.8%)	4 (7.5%)	5 (9.4%)	0 (0%)	1 (1.9%)	0 (0%)
	Microdebrider	0 (0%)	1 (1.9%)	0 (0%)	0 (0%)	0 (0%)	0 (0%)
	Combined (CO_2_ + Microdebrider)	1 (1.9%)	0 (0%)	1 (1.9%)	0 (0%)	0 (0%)	0 (0%)
2021	CO_2_ laser	0 (0%)	2 (3.8%)	2 (3.8%)	0 (0%)	0 (0%)	0 (0%)
	Microdebrider	0 (0%)	1 (1.9%)	1 (1.9%)	0 (0%)	0 (0%)	0 (0%)
	Combined (CO_2_ + Microdebrider)	0 (0%)	2 (3.8%)	1 (1.9%)	0 (0%)	0 (0%)	0 (0%)
2022	CO_2_ laser	0 (0%)	2 (3.8%)	0 (0%)	0 (0%)	0 (0%)	0 (0%)
	Microdebrider	1 (1.9%)	5 (9.4%)	0 (0%)	0 (0%)	1 (1.9%)	0 (0%)
	Combined (CO_2_ + Microdebrider)	0 (0%)	1 (1.9%)	1 (1.9%)	0 (0%)	0 (0%)	0 (0%)
2023	CO_2_ laser	0 (0%)	1 (1.9%)	0 (0%)	0 (0%)	0 (0%)	0 (0%)
	Microdebrider	1 (1.9%)	2 (3.8%)	2 (3.8%)	0 (0%)	0 (0%)	0 (0%)
	Combined (CO_2_ + Microdebrider)	1 (1.9%)	0 (0%)	0 (0%)	2 (3.8%)	0 (0%)	0 (0%)
Total		6 (11.3%)	22 (41.5%)	15 (28.3%)	5 (9.4%)	5 (9.4%)	0 (0%)

## Appendix B

**Table A2:** Patient distribution by age group and outcomes after surgical treatment.

Age Group	CO_2_ Laser—Early Recurrence	CO_2_ Laser—Late Recurrence	CO_2_ Laser No Recurrence	Microdebrider—Early Recurrence	Microdebrider—Late Recurrence	Microdebrider No Recurrence	Combined—Early Recurrence	Combined—Late Recurrence	Combined—No Recurrence
<12 months	1 (10.0%)	1(14.3%)	0 (0.0%)	0 (0.0%)	1 (20.0%)	1 (20.0%)	0 (0.0%)	1 (33.3%)	0 (0.0%)
12–36 months	2 (20.0%)	4 (57.1%)	5 (62.5%)	4 (66.7%)	2 (40.0%)	3 (60.0%)	2 (50.0%)	0 (0.0%)	2 (40.0%)
4–6 years	5 (50.0%)	0(0%)	1 (12.5%)	2 (33.3%)	2 (40.0%)	0 (0.0%)	1 (25.0%)	1 (33.3%)	2 (40.0%)
7–10 years	1 (10.0%)	0 (0.0%)	1 (12.5%)	0 (0.0%)	0 (0.0%)	0 (0.0%)	1 (25.0%)	1 (33.3%)	1 (20.0%)
11–14 years	1 (10.0%)	2 (28.6%)	1 (12.5%)	0 (0.0%)	0 (0.0%)	1 (20.0%)	0 (0.0%)	0 (0.0%)	0 (0.0%)
15–18 years	0 (0.0%)	0 (0.0%)	0 (0.0%)	0 (0.0%)	0 (0.0%)	0 (0.0%)	0 (0.0%)	0 (0.0%)	0 (0.0%)
Total	10 (100%)	7 (100%)	8 (100%)	6 (100%)	5 (100%)	5 (100%)	4 (100%)	3 (100%)	5 (100%)
